# Extreme Variations of pCO_2_ and pH in a Macrophyte Meadow of the Baltic Sea in Summer: Evidence of the Effect of Photosynthesis and Local Upwelling

**DOI:** 10.1371/journal.pone.0062689

**Published:** 2013-04-23

**Authors:** Vincent Saderne, Peer Fietzek, Peter Maria Jozef Herman

**Affiliations:** 1 Benthic Ecology group, GEOMAR: Helmholtz Center for Ocean Research in Kiel, Kiel, Schleswig-Holstein, Germany; 2 Spatial Ecology, NIOZ: Royal Netherlands Institute for Sea Research, Yerseke, Zeeland, The Netherlands; 3 CONTROS Systems & Solutions GmbH, Kiel, Schleswig-Holstein, Germany; University of California, Merced, United States of America

## Abstract

The impact of ocean acidification on benthic habitats is a major preoccupation of the scientific community. However, the natural variability of pCO_2_ and pH in those habitats remains understudied, especially in temperate areas. In this study we investigated temporal variations of the carbonate system in nearshore macrophyte meadows of the western Baltic Sea. These are key benthic ecosystems, providing spawning and nursery areas as well as food to numerous commercially important species. *In situ* pCO_2_, pH (total scale), salinity and PAR irradiance were measured with a continuous recording sensor package dropped in a shallow macrophyte meadow (Eckernförde bay, western Baltic Sea) during three different weeks in July (pCO_2_ and PAR only), August and September 2011.The mean (± SD) pCO_2_ in July was 383±117 µatm. The mean (± SD) pCO_2_ and pH_tot_ in August were 239±20 µatm and 8.22±0.1, respectively. The mean (± SD) pCO_2_ and pH_tot_ in September were 1082±711 µatm and 7.83±0.40, respectively. Daily variations of pCO_2_ due to photosynthesis and respiration (difference between daily maximum and minimum) were of the same order of magnitude: 281±88 µatm, 219±89 μatm and 1488±574 µatm in July, August and September respectively. The observed variations of pCO_2_ were explained through a statistical model considering wind direction and speed together with PAR irradiance. At a time scale of days to weeks, local upwelling of elevated pCO_2_ water masses with offshore winds drives the variation. Within days, primary production is responsible. The results demonstrate the high variability of the carbonate system in nearshore macrophyte meadows depending on meteorology and biological activities. We highlight the need to incorporate these variations in future pCO_2_ scenarios and experimental designs for nearshore habitats.

## Introduction

Human activities since the 19^th^ century led to an increase of atmospheric pCO_2_ from 280 to 392 μatm [1] and the trend is rising. Some scenarios expect an elevation of atmospheric pCO_2_ up to 1000 μatm during the 21^th^ century, peaking around 1400 μatm in the year 2300 [2], [3]. As oceans equilibrate with the atmosphere, dissolution of CO_2_ in water induces a decrease in pH. Global change has already led to an average seawater “acidification” of 0.1 pH units in the world ocean [4]. Acidification is enhancing the corrosiveness of seawater to calcite and aragonite, the two isoforms of calcium carbonates composing the shells and skeletons of marine organisms. Corrosiveness is expressed by the saturation states Ω_arag_ and Ω_calc_. A saturation state below 1 indicates a tendency towards dissolution of the crystal. Aragonitic structures (e.g. scleratinian corals, nacre of bivalve shells) are more soluble than calcitic (e.g. outer shell of oyster) [4]–[5].

Once dissolved, CO_2_ becomes part of the carbonate system, almost entirely composed of bicarbonate (HCO_3_^−^) and carbonate ions (CO_3_^2−^). Under actual atmospheric concentrations of CO_2_, CO_2(aq)_ in the surface ocean represents less than 1% of the dissolved inorganic carbon (DIC) while HCO_3_^−^ represents ∼90% and CO_3_^2−^ ∼10%. The carbonate system in open oceanic environments is well known and most of the data forming the basis for the predictive models (see GLODAP database, [5], [6]) are derived from the open ocean. Those oceanic actual and future pCO_2_/pH values are the ones referred to when designing ocean acidification studies [7]. However, the biogeochemistry of nearshore ecosystems features more variations and differs widely than offshore conditions [8], [9]. Shallow nearshore and estuarine areas are the habitat of numerous benthic calcifiers. As highlighted by Andersson and Mackenzie (2012) [10], investigations on the effects of ocean acidification on calcifiers are neglecting this natural variability.

In nearshore habitats, the few available investigations were conducted on: (1) estuaries, salt marshes, mangroves and mudflats where transfers of carbon from land are occurring (*e.g*. [11]–[13]) and (2) reefs formed by corals or calcifying algae because of the direct effect of calcification on the carbonate system (*e.g*. [14], [15]). The carbonate chemistry of nearshore habitats dominated by macrophytes (kelp forests and seagrass and/or seaweed meadows) is almost unknown, even though they represent ∼5% of the primary production in the ocean and ¾ of the vegetal biomass [16], [17]. In these habitats, the carbonate system is driven by the photosynthetic physiology of the macrophytes, taking up carbon during the day and releasing it by respiration during the night [18], [19]. Since CO_2_ is a minor component of DIC and its diffusion rate in seawater is 10,000 times lower than in air [20], most macrophytes are relying on HCO_3_^−^ for photosynthesis [21]–[24]. Macrophyte meadows are highly productive and net autotrophic (carbon sink) [25], [26] exporting their excess production to the neighboring ecosystems under the form of litter or dissolved organic carbon [27]. Microbial degradation of exported organic carbon leads to elevated pCO_2_ and hypoxia of deeper offshore waters [28]. This phenomenon is particularly important in eutrophied ecosystems like the Baltic Sea [29]. In the inner bays of the western Baltic, local upwelling of hypercapnic water masses is regularly observed, increasing the surface pCO_2_ up to 2500 μatm. This typically happens at the end of summer when offshore winds are upwelling deep waters after long periods of stratification [30]–[32].

The aim of this study, conducted in the western Baltic Sea over three weeks of summer 2011, was to measure (1) the day/night variations of pCO_2_ due to photosynthesis and respiration of a macrophyte bed, (2) the evolution of the baseline pCO_2_ over summer and (3) the effect of local upwellings on the carbonate system and its diel variations. The variations of DIC, measured in the macrophyte meadow, were modeled with a statistical model considering wind direction and speed together with photosynthetically active radiation (PAR).

## Materials and Methods

### 2.1 Study site

**Figure 1:**
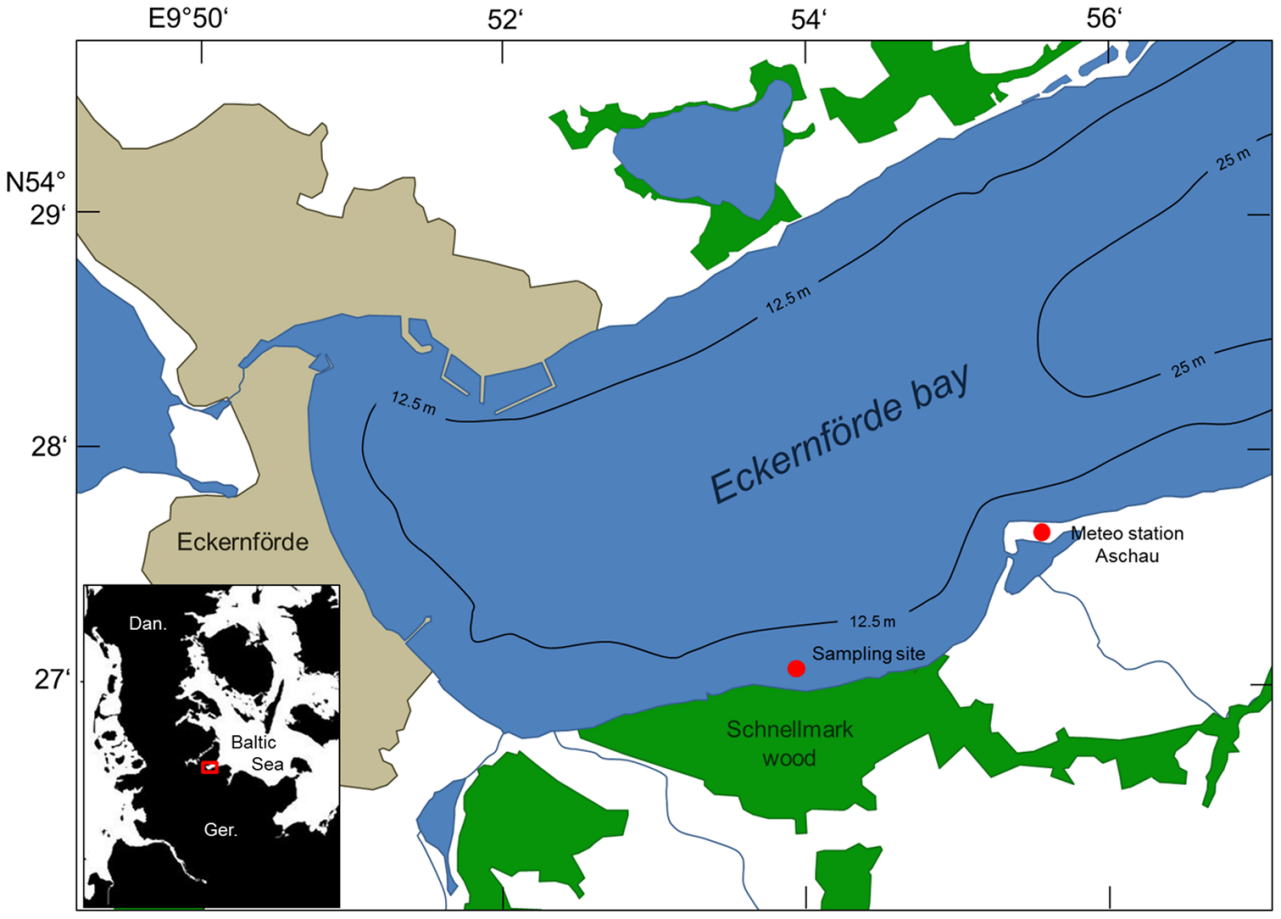
Study site. Map of the inner Eckernförde Bay indicating the locations of the survey site and the meteorological station, urban area (brown) and forests area (green).

Physico-chemical parameters of seawater were recorded in a macrophyte meadow (3 m depth) in Eckernförde Bay (western Baltic Sea, Germany, 54°27′ N, 9°54′ E, see Fig. 1), during 3 weeks of summer 2011: July: 29.06−08.07, August: 29.07–05.08 and September: 09.09–16.09. In July, only pCO_2_ and PAR irradiance were recorded by two independent sensors. For the August and September deployments, a multimeter recording pH, salinity and temperature was added to the CO_2_ and PAR sensor. Wind speed (m s^−1^) and direction (rad) with a resolution of 10 min were provided by the meteorological station of Aschau in Eckernförde Bay (54°27′40 N, 9°55′30 E) belonging to the division of marine technology and research of the German Navy. The nearshore habitat of Eckernförde is a mixed bottom type dominated by macrophyte vegetation. The macrophyte vegetation covers approximately 75% of the surface. Dominant species are the brown algae *Fucus serratus* (40–60% of the macrophytes), growing on stones and rocks and the seagrass *Zostera marina* (<10% of the macrophytes) growing on sandy bottoms [33]. The inner basin reaches a maximum depth of 28 m in gentle slope. The water column is stratified in summer, with a pycnocline around 15 m depth [34]. September is the period of maximal stratification and hypoxia in the deep water. Hence, O_2_ reaches minimal concentrations of about 30 μM, associated with peaks of pCO_2_ up to 2500 μatm and accumulation of dissolved methane [29], [35]. No specific permits were required for the study, the location is not privately-owned or protected in any way and the study did not involve endangered or protected species.

### 2.2 The autonomous measuring system

The pCO_2_ was measured with a HydroC™ underwater sensor (CONTROS Systems & Solutions, Germany). The instrument measures the CO_2_ mole fraction in a headspace behind a membrane equilibrator with a two-wavelength non-dispersive infra-red detector (NDIR). The equilibrator is composed of a flat silicone composite membrane, the NDIR unit and additional sensors for pressure, temperature and relative humidity used to correct the NDIR signal and calculate the partial pressure. A small internal pump insures the continuous circulation of air in the equilibrator. To correct the drifting of the instrument with time, regular zeroings are automatically performed by scrubbing the CO_2_ from the internal gas stream. The zeroings are accounted during post processing of the data. A first description of the instrument can be found in [36] and deployments on various platforms are presented in [37]. The instrument was calibrated by the manufacturer prior to every deployment in the range of 100–1000 µatm at 17°C, 18°C and 18°C for the first, second and third deployment respectively. An overall accuracy of better than ±1% of the upper range value is assumed for post processed data. In order to achieve a constant and sufficiently short response time of the instrument, the HydroC™ is equipped with a water pump (Sea-Bird Electronics, USA) that provides a continuous water flow of approx. 35 ml sec^−1^ to the membrane, leading to a response time T_63%_ of 3 min and a T_99%_ of approximately 15 min. These response times are sufficiently fast to resolve the observed signal changes without the need for signal deconvolution. To correct the pCO_2_ series for the instrument drift, the zerooing signals were regarded as nodes and linearly interpolated in time to result in a smooth signal drift correction throughout the deployment time.

Salinity (psu), voltage (analogue signal from the pH measurement) and temperature (°C) were recorded automatically every 45 min with a pH-meter/conductimeter Mettler-Toledo SG 7/8 (Mettler Toledo, Switzerland). For the measurement of pH on total scale, seawater TRIS pH buffers for 15 psu were made according to the SOP 6a of Dickson et al. (2007) [38]. A combined electrode with a solid polymer electrolyte reference, equipped with an NTC (negative temperature coefficient) thermistor was used (Inlab expert pro, Mettler Toledo, Switzerland). The calibration of a new electrode was made 24 h before each deployment. To do so, the TRIS buffer was immersed in a thermostatic bath and the voltage of the pH electrode was measured with an accuracy of ±0.1 mV. The temperature of the buffer was varied by 1.5°C around seawater temperature. The temperature corresponding to every mV change was recorded with accuracy better than 0.01°C with a Fluke 5658 reference thermometer doted of a 5608 platinum resistance sensor (Fluke, USA). This process was repeated by increasing and decreasing the temperature to get an average voltage (mV) *versus* T (°C) reference curve for the electrode in the buffer. The NTC sensor of the pH sensor, of resolution 0.1°C, was calibrated against a reference thermometer and the resulting regression was achieved with a R^2^ >0.99. The resulting equation was used to correct the sampled temperature. The sampled voltage and corrected temperature were converted to pH_tot_ by making use of the initial TRIS buffer calibration of the electrode and by using the equations given in the SOP 6a. In the lab, work conducted on Certified Reference Material (CRM) (Andrew Dickson, Scripps Institution of Oceanography) together with 35 psu buffers demonstrated an accuracy of 0.003 to 0.005 pH units and a precision better than 0.001 pH unit. However, this accuracy was not reached in the field and therefore the pH series were not used to derive the carbonate system but instead the alkalinity from discrete samples (see 2.3 for method and 4.4 for discussion on the method).

Salinity was measured within 0.01 psu by a Mettler Toledo Inlab 738 conductivity probe after calibration at 25°C with KCl 0.1 mol L^−1^ (Fischer Scientific, USA). The PAR irradiance (400 to 700 nm) was recorded every 5 min using a LI-192 quantum sensor connected to a LI-1400 data logger (Li-Cor Biosciences, USA). All series were extended to one minute interval series by linear interpolation.

### 2.3 Calculation of the carbonate system

Samples for alkalinity were taken at the beginning, middle and end of the measurement periods of August (the 29.07, 02.08 and 05.08, no replication) and September (the 09.09, 13.03 and 16.09, one replicate). Alkalinity was measured with an accuracy of ±5 µmol kg^−1^ using an open cell potentiometric titrator as described in the SOP 3b of [38] (Titrando 888, Methrom, Switzerland). A regression was calculated between the total alkalinity and salinity of the samples from August and September pooled together. The regression was highly significant (F-statistic: p<0.001, R^2^  = 0.83, n = 9): Alkalinity  = 905.17 * Salinity +59. 83, with both slope and intercept significant at p<0.001 and p<0.01 (t-test, n = 9). Therefore, the alkalinity for the entire period was estimated from the salinity series. The DIC, Ω_arag_ and Ω_calc_ were derived from calculated alkalinity and the measured pCO_2_ according to the equations described in [39] with the R package Seacarb [40] using first and second carbonate system dissociation constants for estuarine systems from [41] and the dissociations constants of HF and HSO4^−^ of [42], [43].

### 2.4 Statistical modeling

The calculated DIC series of August and September was explained through a statistical model. It considered that (1) the weekly trend of the DIC series is caused by the upwelling of DIC-rich bottom water, (2) the diel variation of the DIC series is caused by primary production and respiration of the meadow. The weekly trends of DIC (Cw) were separated from the diel oscillations (Cd) by a central running average with a time frame of 24 h. Upwelling was assumed to be driven by wind. Cw was fit by a function of wind speed weighted by wind direction (WWt).
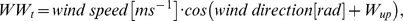
With W_up_ being a parameter between 0 and 2π, corresponding to the wind direction for which wind-induced upwelling is maximal. As examples, if W_up_  = 0 or 2π, maximal weights are given to northern winds, if W_up_  =  π maximal weights are given to Southern winds. A “left-sided” running integration was performed on the weighted wind time series over a period k_w_:



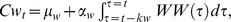



Where Cw_t_ is the 24 h running-average series of DIC at time t, WW_t_ is the weighted wind speed at time t, μ_w_ and α_w_ are regression parameters and k_w_ is determined as the integration period yielding the best fit. Parameters in the regression were chosen to minimize residual variance. In the regression between time series, formal hypothesis tests, F-statistics and regression coefficients were not considered as they would be biased due to auto-correlation.

The within-day variations of DIC, Cd were modeled by exponentially weighted running integration of the PAR series over a period of 12 hours with an exponential decay rate λ (min^−1^):
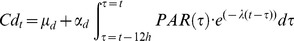



In a final step, the irradiance and wind sub-models were summed to obtain the final model. The standard deviation of the residuals between model and DIC observation were considered for the parameterization of W_up_, k_w_ and λ.

## Results

### 3.1 Measures

The three weeks revealed important day/night oscillations of pH and pCO_2_. The pCO_2_ increased during the night and decreased during the day reaching minima and maxima at 18∶00 and 6∶00 respectively. The pH inversely mirrored these pCO_2_ variations.

**Figure 2:**
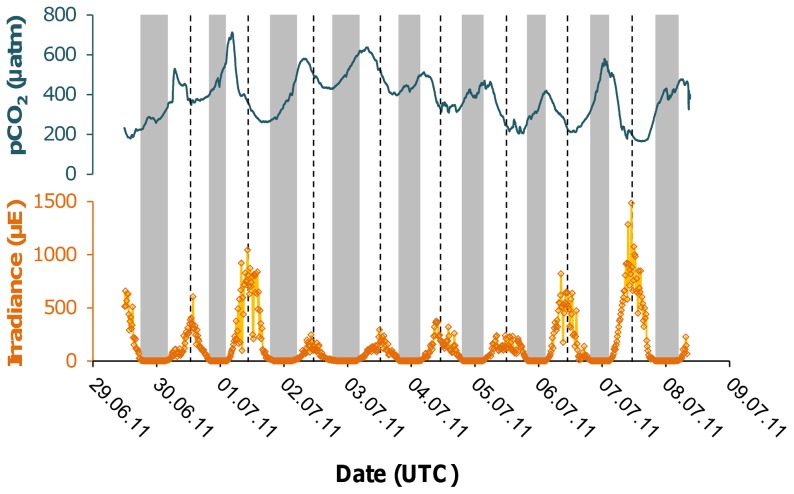
Comparison between Light and pCO_2_ series in July. Dark bands: period of darkness. Dashed lines: estimated center of the daylight distribution.

In July, only pCO_2_ and light was recorded. The mean pCO_2_ of the week was 390 μatm (mean ± SD), corresponding to the atmospheric CO_2_. However, diel oscillations of 281±88 μatm (mean ± SD) were recorded, with a phase shift of ∼6 h between light and pCO_2_ cycles. This shift, common to all three measurement periods is illustrated for July in Fig. 2.

**Figure 3:**
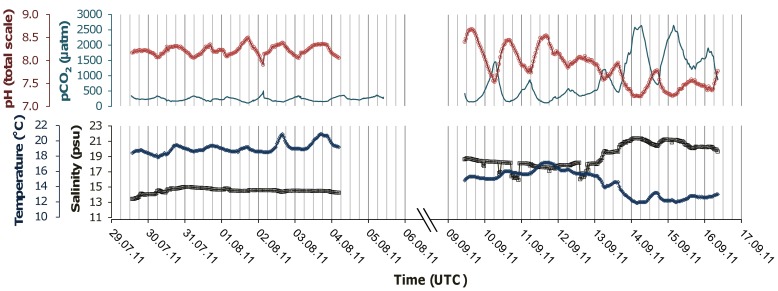
In situ measurements for August and September. pCO_2_ (cyan), pH (red) Salinity (black) and temperature (dark blue) recorded in August (left) and September (right). The recording frequency was one minute for pCO_2_ and 45 minutes for the 3 other parameters. Symbols in the plots mark the recording events; linkage between the measurements every 45 min was achieved by linear interpolation. Secondary vertical gridlines unit: 6 h.

In August, seawater was undersaturated for CO_2_ compared to the atmosphere with a week mean of 239 μatm only. However, the diel variations remained comparable to those of July with a mean of 219±24 μatm (± SD) (Fig. 3, top left panel). The mean ± SD daily maximal and minimal pCO_2_ values were 374±67 and 155±32, respectively. The maximal amplitude was 416 μatm. The pH variations mirrored fluctuations in pCO_2_ with a weekly mean of 8.21, mean diel amplitude of 0.34±0.15 (± SD) and mean daily minimum and maximum of 8.03±0.07 and 8.37±0.08 (± SD). The average salinity and temperature during the week were 14.5±0.3 psu and 19.1±0.7°C, respectively (Fig. 3, lower left panel).

The recordings of September exhibit a strong difference between the beginning (09.09 to 13.09) and the end of the week (13.09 to 16.09). The mean ± SD salinity and temperature during the first three days were 17.8±0.6 psu and 15.9±0.6°C while during the three last days they increased and decreased to 20.7±0.5 psu and 12.4±0.4°C respectively (Fig. 3, lower right panel). This sudden decrease in seawater density is revealing an upwelling occurring in the middle of the week. Consequently, we observed a large discrepancy of the pCO_2_/pH between those two periods with means of 426 μatm/8.14 and 1593 μatm/7.46 respectively (Fig. 2, top right panel). In that second part of the week, oscillations of pCO_2_ became extreme. The maximal daily amplitudes recorded were 2184 μatm pCO_2_ and 1.15 pH units. The night peaks (mean ± SD) of pCO_2_ were 2397±425 μatm, with drops of pH to (mean ± SD) 7.26±0.07. During daytime, minimum pCO_2_ levels were still much higher than atmospheric concentrations with minima of (means ± SD) 681±211 μatm and corresponding maxima of pH of 7.77±0.18.

### 3.2 Carbonate system

**Figure 4:**
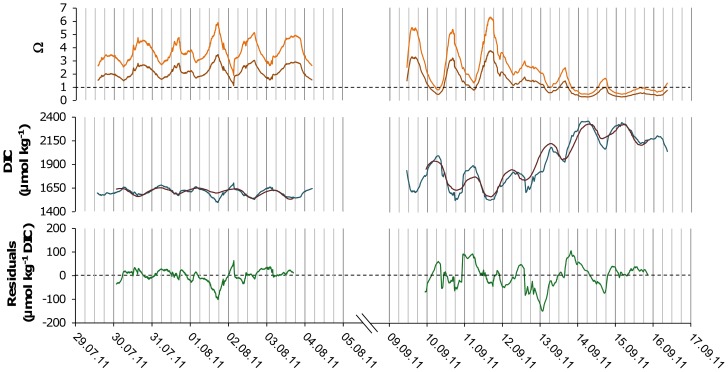
Calculated and modeled carbonate chemistry. Top panels: Saturation states for calcite (light brown) and aragonite (dark brown) for August (left) and September (right). Dashed line: Saturation threshold. Central panels: Observed (cyan) and modeled (red) DIC for August (left) and September (right). The residuals between model and observations are presented in bottom left and right for August and September respectively. Secondary vertical gridlines unit: 6 h.

In August, means ± SD of the week and day/night variations of DIC were 1609±37 μmol kg^−1^ and 131±45 μmol kg^−1^ respectively (Fig. 4 central left panel). The seawater was always supersaturated with respect to both aragonite and calcite with diel means ± SD of 2.2±0.2 and 3.7±0.3 and means ± SD day/night variations of 1.4±0.6 and 2.4±0.9 respectively (see Fig. 4, top left panels).

In September the daily mean ± SD DIC concentrations preceding and during the upwelling event were 1829±45 and 2158±27 μmol kg^−1^, respectively (Fig. 4 central right panel). The diel variations of DIC were higher before than during the upwelling, with mean ± SD of 259±10 μmol kg^−1^ against 205±91 μmol kg^−1^, respectively.

Before the upwelling, the seawater was almost always supersaturated for aragonite and calcite (see Fig. 4 top right panel, 09.09 to 13.09). However at the hours of maximal pCO_2_ (6∶00), aragonite saturation dropped below 1: mean Ω_arag_: 0.8±0.3 (± SD). Oppositely, seawater reached maxima for Ω_arag_ and Ω_calc_ around 18∶00. The resulting diel oscillations were of 4.3±0.3 and 2.6±0.2 for calcite and aragonite, respectively.

During the upwelling event the seawater was constantly undersaturated with mean ± SD Ω_calc_ and Ω_arag_ of 1±0.5 and 0.6±0.3, exception made of the hours around 18 h, were modest maxima (mean ± SD) of 1.9±0.6 and 1.1±0.4 were reached (see Fig. 4 top right panel, 13.09 to 16.09).

### 3.3 Model

The DIC calculated from pCO_2_ and alkalinity and the DIC predicted by the statistical model for August and September are presented in Fig. 4 central panels. The differences between both are presented in Fig. 4. lower panels (residuals). In August, the model predicted a diel DIC mean (± SD) of 1610±16 µmol kg^−1^ and diel amplitudes of DIC of 86±14 µmol kg^−1^ (mean ± SD). The differences between modeled and calculated DIC is 1 µmol kg^−1^ for the diel average and 44 µmol kg^−1^ for the diel amplitude.

In September, the model predicted a diel DIC mean (± SD) of 1852±83 µmol kg^−1^ before the upwelling and 2143±47 µmol kg^−1^ during the upwelling. The differences with the calculated DIC are 23 µmol kg^−1^ and 15 µmol kg^−1^, respectively. Before and during upwelling, the diel amplitudes of DIC predicted by the model are 141±61 µmol kg^−1^ and 150±107 µmol kg^−1^ (mean ± SD), respectively. The difference from the calculated DIC is 117 µmol kg^−1^ and 55 µmol kg^−1^.

**Figure 5:**
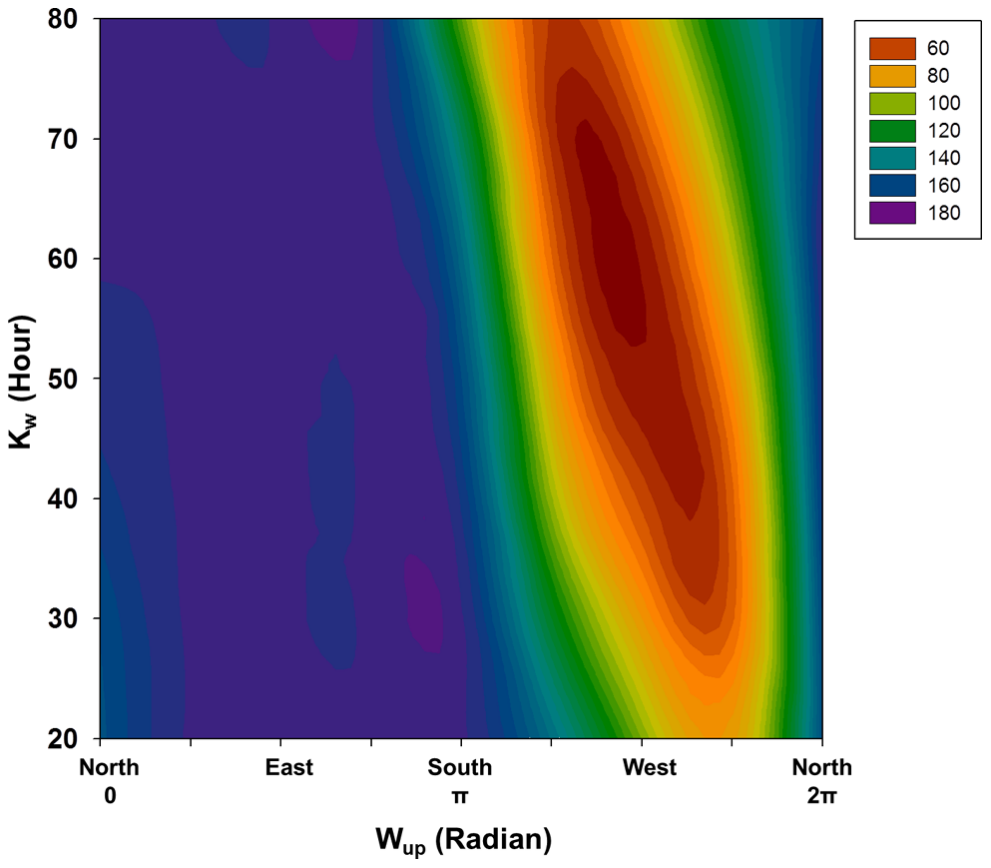
Sensitivity of the model to the integration period k_s_ and wind direction W_up_. Contour plot of the standard deviation of the residuals between model and observation in µmol kg^−1^ DIC (color palette) for the September series as a function of W_up_, the azimuth of reference used as maximal weight, and the k_w_, the period of the running integration of the weighted wind series.

**Figure 6:**
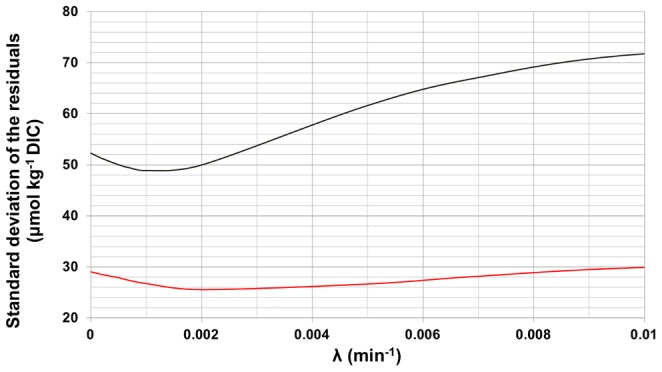
Sensitivity of the model to λ. Evolution of the standard deviation of the residuals between model and observation in µmol kg^−1^ DIC in August (red) and September (black) as a function of λ (min^−1^), the instantaneous rate of increase used for the exponentially weighted running integration of the irradiance series.

The set of parameters producing the best fitting models are W_up_  = 3π/2, corresponding to westerly wind, a period of integration k_w_ of 55 h and λ of 0.0025 min^−1^and 0.001 min^−1^ for August and September respectively. The standard deviation of the residuals between modeled and calculated DIC are 25 μmol kg^−1^ and 49 μmol kg^−1^, corresponding to percentages of unexplained variation of 1.6% and 2.5% for August and September respectively (Fig. 3 lower panels). Fig. 5 presents, for September only, the evolution of the model residuals as function of the wind direction (W_up_) and the integration period (k_w_). Easterly winds as maximal weights produce the worst fitting models while westerly winds the best fitting ones, southerly and northerly winds are intermediate. The parameter λ had less influence on the outcome of the model (Fig. 6), increasing its accuracy by a maximum of 2 to 4 μmol kg^−1^. Despite its poor effect on the model, the presence of this parameter is justified by the consideration of the decrease of the day length between August and September (the period of integration of the irradiance is fixed to 12 h in both).

## Discussion

In our study, we showed the importance of the diel variations of pCO_2_ and pH due to photosynthesis and the importance of the variations of the carbonate chemistry baseline over summer. We demonstrated the interactive effects of upwelling and algal metabolism on the carbonate chemistry with a simple statistical model.

### 4.1 Inter-weekly dynamics

The pCO_2_ of our measurement site exhibited very different weekly trends over the summer. In August, we observed an important and stable pCO_2_ undersaturation. This reflects the conditions of the whole Baltic at this period, generated by the succession of blooms of phytoplankton and cyanobacteria [44], [45]. In September this stable condition is interrupted by the strengthening of the westerly winds, leading to the upwelling of the water masses isolated until then below the pycnocline. The chemistry of the deep waters of Eckernförde bay are monitored since 1957 and pCO_2_ of about 2500 μatm, linked to heterotrophic processes, are yearly observed in summer (Boknis Eck time series: [46]). Our study shows that the nearshore biota in shallow water is exposed to these high concentrations during upwelling events. The deep-water pCO_2_ is presumed to increase in the future by the conjunction of higher atmospheric pCO_2_ and reduced salinity and alkalinity of the Baltic Sea [32]. Melzner et al. (2012) [29] are expecting this deep water body to reach pCO_2_ of 4000 to 5000 μatm and the hypercapnic events of September to increase in magnitude. Besides, the frequency and duration of those upwelling events could also increase as a reinforcement of the westerly winds in the Baltic region is expected with global warming [47].

### 4.2 Daily oscillations

Diel oscillations, related to photosynthesis and respiration, are superimposed to the week scale dynamics. In normal summer conditions (without upwelling), the mean (± SD) amplitudes of the diel variations were 243±95 μatm CO_2_ (July, August) and 0.34±0.15 pH units (August only). Such diel variations have already been observed in macrophyte stands worldwide: in seagrass beds of Australia [16], Mediterranean [48] and Zanzibar [19], in tidal rocky-shores of the northeastern Pacific [49] and in algal meadows of the Danish islands [18]. Among nearshore ecosystems, the highest diel variations are occurring in macrophyte dominated ecosystems, upwelling areas and estuaries [50]. Our study site cumulates those three characteristics: brackish, weakly buffered and eutrophied ecosystem dominated by macrophytes and submitted to upwelling. We recorded diel oscillations of 1604.9±795.7 μatm (mean ± SD) during the upwelling event of September. To our knowledge only near-shore mangroves exhibit wider diel variations with 3500 μatm recorded in the Bermuda [10].

However, the importance of these variations is relative, as the fraction of DIC present as CO_2(aq)_ in high pCO_2_/low pH seawater is higher than in low pCO_2_/high pH seawater (see Bjerrum plot in *e.g*. [39]). Thus, in September, the diel variations of CO_2_ are stronger during the upwelling than before but we observe the opposite for DIC (mean ± SD DIC: 258.6±10.5 μmol kg^−1^ against 205.1±90.7 μmol kg^−1^), possibly revealing a reduction of photosynthesis during the upwelling. This could be explained by an osmotic stress engendered by a rapid increase of salinity [51], [52] rather than by the elevated pCO_2_. Indeed, any increase in DIC and pCO_2_ is presumed to be beneficial to marine macrophytes [53], even if this have never been formally proven [54].

### 4.3 Model

We were able to explain the DIC variations of August and September to an accuracy of±50 μmol kg^−1^ with a simple statistical model (three parameters only), based on wind and PAR. The weighing procedure of the wind speed, although simple in its geometric approach, appeared to work very well as an explanation of the multi-day trends.

Our model designates westerly winds as responsible for upwelling. This result is slightly different from previous observations and simulations in other regions of the Baltic Sea identifying southwesterly winds as being the most effective [30]. This small discrepancy is certainly due to the east-west orientation of Eckernförde bay compared to the general north-east/south-west orientation of the Baltic Sea. The use of exponentially weighted integration of irradiance as a proxy for primary production was less effective than the similar approach used for the upwelling. Indeed, while the daily DIC means are quite accurately predicted (<20 µmol kg^−1^), the diel amplitude is systematically underestimated by 40 to 70 µmol kg^−1^ DIC. This variation, unexplained by the model, could be due to heterotrophic respiration, air-sea exchange of CO_2_ [17] or diel variation of alkalinity as discussed in 4.1. However, despite the simplistic nature of our model, it is a first step in the understanding and prediction, in a context of global change, of the carbonate system dynamics in the Baltic nearshore areas.

### 4.4 Measurement reliability

The accuracy of the post-processed pCO_2_ data is expected to be <±10 μatm for values within the calibrated measuring range of 100–1000 µatm. An additional error can be expected when the pCO_2_ exceeds the measuring range as in this case the calibration polynomial of the instrument is extrapolated. We expect this error to be ±150 μatm at maximum for the highest pCO_2_ recorded in September at around 2600 μatm.

Despite our efforts, we did not achieve in the field the pH accuracy necessary to use it as input parameter for the derivation of the carbonate system, as we are able to in laboratory. We estimate the error in pH of the order of 0.01 due to uncorrectable drift during the measurement periods. In the conditions of temperature and salinity of the western Baltic, such inaccuracy in pH produces anomalies in derived alkalinity of the order of 10–100 μmol kg^–1^ at pH/pCO_2_ inferior to 7.8/800 μatm and up to 1000 at higher pH/lower pCO_2_. For future field studies, Durafets sensors [55] or spectrophotometric sensors [56] represent promising alternatives to glass electrodes, both capable of reaching accuracies of 0.001 to 0.0001 pH units. We had to perform alkalinity titrations and rely on a salinity to alkalinity relationship to achieve the calculations. This method is relevant [57], [58] and we estimate the error on the derived DIC to be <15 µmol kg^−1^ DIC. However, it ignores any changes of alkalinity at constant salinity. This phenomenon is very important in coral and shellfish reefs due to the uptake or release of Ca^2+^ by calcification or dissolution [59], [60]. However, in macrophyte ecosystems, we expect very marginal diel variations of alkalinity due to photosynthesis and respiration [61].

### 4.5 Effect on fauna

The daily oscillations of pCO_2_ generated by photosynthesis could be of prime importance for calcifiers, creating at daytime periods of high saturation states favourable to CaCO_3_ precipitation. Such coupling between photosynthesis and calcification has already been observed in a Hawaiian coral reef by Drupp et al. (2011) [59] and Shamberger et al. (2011) [60] where calcification is maximal at midday when the pCO_2_ is minimal due to planktonic photosynthesis. In that reef, the intensity of the photosynthesis is modulated by wind driven inputs of nutrients from the flume of a neighboring estuary.

In general, studies conducted on western Baltic populations of animals, calcifying or not, tend to demonstrate their tolerance to acidic conditions [62]. Also, the Baltic population of the mussels *Mytilus edulis* experiences reduced growth and dissolution of the shell only when Ω_arag_ ≤0.15 corresponding to a pCO_2_ of ∼ 4000 μatm [31]. Despite this weakening of their shells, the predation by sea stars *Asterias rubens* and crabs *Carcinus maenas* maintained at similar pCO_2_ are reduced by 56% and 41% respectively [63]. Besides, the growth of the Baltic brackish barnacle *Amphibalanus improvisus*, competitor of *Mytilus* for space, remains unaffected at both larval and adult stage at pCO_2_ >3000 μatm [64]. Macrophyte meadows are also transient habitats, sheltering early life stages of numerous animal species. Those might exhibit more tolerance to ocean acidification as well. As example, the spawns of Baltic herrings *Clupea harengus*, deposited on macrophyte beds [65], are not affected in their embryonic development by high pCO_2_/low pH [66]. The volatility of the carbonate system and the extreme acidic events we observed could have exerted a selective pressure on Baltic populations, explaining the resistance of the local fauna to acidification stress in laboratory. Nevertheless, all the studies quoted previously were conducted under stable elevated pCO_2_. None coupled elevated pCO_2_ baseline and the diel variations we observed.

## Conclusion

Our study represents one of the first attempts of high resolution continuous measurement of the carbonate system in the highly variable environment that is the Baltic Sea's nearshore. The three weeks showed quite different results related to the dynamics of the whole Baltic carbonate system, to the meteorological condition and to very local processes of photosynthesis and respiration. This study highlights the importance of the natural variations of pCO_2_ and pH and emphasizes the consideration of these in ocean acidification studies on nearshore organisms.
